# Study on elastic-plastic displacement response spectra of base-isolated structures and its influencing factors

**DOI:** 10.1371/journal.pone.0341723

**Published:** 2026-03-13

**Authors:** Tianni Xu, Yiming Wang, Siru Qian, Qin Meng, Shuyu Guo

**Affiliations:** Chengdu Industrial Vocational Technical College, Chengdu, China; Niloufer Hospital, Institute of Child Health, INDIA

## Abstract

In seismic design, the acceleration response spectrum method is used to calculate the bearing capacity of structures to ensure their safety. However, under intense earthquake action, insufficient deformation capacity of structures is the main cause of their failure. For base-isolated structures, the failure is caused by the displacement of the isolation layer exceeding the limit value. To derive practical elastic-plastic displacement spectra for base-isolated structures and incorporate them into seismic design, this paper categorizes 1217 strong motion records, with peak accelerations exceeding 10 gal, into nine groups based on soil conditions and earthquake type. To make the obtained displacement response spectra more accurate, this paper establishes the motion equation of a two-degree-of-freedom system model, using non-proportional damping and the Bouc-Wen model to describe the damping and mechanical characteristics of the isolation layer, the elastic-plastic displacement response spectrum is calculated using numerical methods. Accordingly, a practical formula for the elastic-plastic displacement spectra of base-isolated structures is established by analyzing the factors influencing (soil conditions, earthquake type, parameters in Bouc-wen model) the spectra. Herein, the elasto-plastic displacement response spectrum for base-isolated structures was established, and a formula for calculating the displacement of the isolation layer in base-isolated structures with natural vibration periods within 10 seconds was provided, along with the design peak ground displacement under different conditions. Based on the elastic-plastic analysis of two distinct base-isolated structures, results demonstrate that the current formula is an effective and rapid predictor for estimating the displacement of the isolation layer in base-isolated structures considering various site conditions.

## 1. Introduction

In the current seismic design code in China [1], the horizontal seismic action of structures is calculated using the acceleration response spectrum. Designers believe that as long as the structures meet the requirements of bearing capacity, their safety can be ensured. However, as researchers have gradually deepened their understanding of the elastic-plastic response of structures, it has been discovered that controlling the performance of structures solely based on bearing capacity does not guarantee their safety. This is because the failure of structures under intense earthquake loads does not depend on the instantaneous seismic action, but rather on insufficient deformation capacity, which is the main cause of structural collapse [2,3].For base-isolated structures, the structural displacement under seismic action mainly concentrates in the isolation layer, and the variation of displacement in the isolation layer has a significant impact on the isolation effect. Controlling the displacement of the isolation layer is the key to ensuring structural safety and good seismic performance. Therefore, in the seismic design of base-isolated structures, not only the acceleration response spectrum is used for seismic response calculation, but also the displacement response spectrum is used to verify the maximum displacement response of the structure. Based on this, displacement-based seismic design, as an important aspect of performance-based seismic design methods, has attracted wide attention from domestic and foreign scholars [2–5]. From an engineering perspective, research has been conducted on the elastic-plastic displacement response spectrum of structures, Shi Weixing [6] fitted the acceleration and displacement response spectra for the design of base-isolated structures and presented simplified expressions for both spectra. Considering the errors in the long-period segment of the design response spectrum, Huang Hairong [7] proposed a calculation formula for the response spectrum suitable for base-isolated structures and verified its reliability through numerical examples. Cao Jialiang [8,9] compared the differences between the pseudo-spectra and real spectra of base-isolated structures. By selecting appropriate strong motipn records, they established relative displacement, relative velocity, and absolute acceleration response spectra corresponding to the code-specified fortification intensity for a certain range of damping ratios and periods. These spectra can be used to predict structural responses. Reference [10] established a design displacement spectrum for base-isolated structures, providing a method for displacement-based seismic design. Using the equations of motion for a simplified two-degree-of-freedom model of a base-isolated structure, Teng Jun [11] analyzed the amplitude-frequency characteristics of the displacement transfer function of each mass and proposed a method to optimize the damping ratio and stiffness coefficient of the isolation layer, along with simplified parameter calculation formulas for practical engineering applications. Based on the elastoplastic response spectrum theory and referring to the relationship between the equivalent damping ratio and displacement ductility in nonlinear restoring force models, He Wenfu [12] established an elastoplastic response spectrum for base-isolated structures. The accuracy of the proposed elastoplastic response spectrum for seismic response analysis of base-isolated structures was verified through shaking table tests, numerical simulations, and design response spectrum calculations of a five-story steel frame base-isolated structure. Qamaruddin [13] simplified a brick-concrete isolated structure into a two-degree-of-freedom model and obtained its acceleration response spectrum, sliding displacement spectrum, and residual displacement spectrum using the El Centro and Koyna shock earthquake waves as excitation. The influence of different parameters of the simplified model on the dynamic characteristics of the isolated structure was studied through the response spectra. Ye Kun [14,15] equivalent the base-isolated structure to a two-degree-of-freedom (2DOF) model. Then, through the equivalent linearization of the LRB isolation system and the mode decomposition response spectrum method, they established analytical expressions for the displacement response of the isolation layer and superstructure in the equivalent 2DOF model and their relationship with structural parameters. A unified seismic design method for base-isolated structures based directly on displacement design (DDBD) was proposed, and the effectiveness of this method was verified by time-history analysis results. then derived simplified calculation formulas for the mechanical performance parameters of the LRB isolation system, the seismic action on the superstructure, and the maximum horizontal deformation of the isolation layer based on the direct design method and the mode decomposition response spectrum method. Through design examples, they evaluated the seismic performance of LRB base-isolated structures designed using the direct design method and verified the effectiveness of the reasonable range of mechanical performance parameters determined for the isolation system. Hu jinjun [16] proposed prediction equation can be applied to anticipating the displacement response of self-centering systems in near fault area.In order to ensure the safety of base-isolated structures, different methods were adopted to study the seismic performance of base-isolated structures [17–22].

On the one hand, the design response spectrum in the Code for Seismic Design of Buildings is an elastic response spectrum for non-isolated structures. Although it can effectively represent the elastic response of structures under “minor earthquakes”, as the earthquake intensity increases, the isolation layer of base-isolated structures will yield first, the response of the isolation layer cannot be obtained through the design response spectrum [23]. Therefore, the pseudo-displacement response spectrum obtained from the code response spectrum cannot effectively calculate the displacement of the isolation layer [24]. Reference [25] points out that due to the high damping and concentrated deformation of the isolation layer, it is necessary to not only use the acceleration spectrum for seismic design but also check its displacement. Therefore, a displacement response spectrum and calculation formula suitable for base-isolated structures are proposed.

On the other hand, the isolation design of base-isolated structures aims to ensure that the horizontal displacement of each isolation bearing in the structure under rare earthquakes meets the limit horizontal displacement, the seismic code of China only mentions that “the ultimate horizontal displacement of the isolation bearing under different building categories corresponding to the compressive stress should be greater than 0.55 times the effective diameter of the bearing and the maximum value of three times the total thickness of the internal rubber.”. The isolation gap is set according to the maximum horizontal displacement of the isolation bearings. However, studies have found that site condition and earthquake type have certain effects on the spectral values of the displacement spectrum [26], which should be particularly noted in calculations. Meanwhile, the maximum displacement of the isolation layer is determined through time-history analysis, which requires selecting multiple ground motion records and is computationally intensive and time-consuming. Therefore, considering the convenience of response spectra, it is necessary to propose a nonlinear displacement spectrum for the isolation layer that accounts for factors such as site category, fault distance, and non-proportional damping characteristics of the base-isolated structure. This spectrum can not only effectively solve the problem of heavy computation and time consumption but also integrates multiple influencing factors, enabling convenient and rapid estimation of the maximum displacement of the isolation layer and the width of the isolation gap under rare earthquakes, thereby guiding the design of the isolation layer. Therefore, it is necessary to study the nonlinear displacement spectrum of the isolation layer in base-isolated structures.

In this study, 1217 strong motion records were selected, and they were grouped according to the site classification criteria proposed by Lv Hongshan [27]. The simplified model of a two-degree-of-freedom base-isolated structure was directly subjected to elastic-plastic time-history analysis under seismic action, a characterization model for the isolation layer spectrum considering non-proportional damping characteristics is proposed during the calculation process, which corrects the shortcomings of the traditional proportional damping assumption; meanwhile, three influencing factors of the elastic-plastic response spectrum of this structure were studied [28–31]. Finally, the a three-segment elasto-plastic displacement spectrum calculation formula of the modeled displacement spectrum for predicting the maximum displacement of the isolation layer of the base-isolated structure and the maximum values of the displacement of the isolation layer under different conditions were obtained. This allows for the rapid and accurate derivation of the displacement of the isolation layer in base-isolated structures.

## 2. Strong motion records

**Fig 1:**
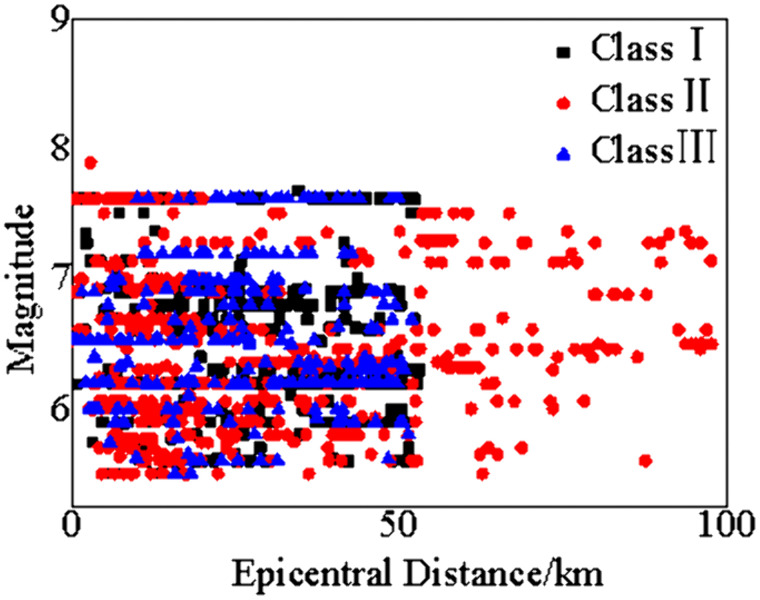
Distribution of magnitude and epicentral distance for 1217 strong motion records.

Extensive studies have shown that response spectra are directly influenced by site conditions [32–34]. Seismic codes stipulate that sites for base-isolated structures should be classified into Site Classes I, II, and III. Near-field ground motions have significantly different characteristics from far-field ground motions and exert a more severe impact on buildings. Therefore, to improve the applicability of the obtained response spectra for base-isolated structures, 1217 strong ground motion records were finally selected from the Pacific Earthquake Engineering Research Center (PEER) in the United States for data analysis, following the “multi-criteria hierarchical selection method” proposed by Caicedo et al. [35] and considering earthquake magnitude, site class, fault distance, pulse characteristics, and earthquake mechanism. The specific classification is shown in Table 1 and Fig 1. Table 2 presents the coefficients of variation (CV) of peak ground acceleration (PGA) for nine groups of strong motion records, showing that the CV values range from 0.74 to 0.89. This dispersion is a result of “intentionally retaining data diversity,” aiming to cover more engineering scenarios and improve the adaptability of response spectra.

**Table 1:** The selected strong motion records.

Site Classes	Class I	Class Ⅱ	Class Ⅲ
Near-field pulse (NF)	25	67	26
Near-field non pulse (NFN)	113	234	87
Far-field (FF)	207	289	169

**Legend:1.Site classification is based on the Ref [11], using the equivalent shear wave velocity (Vs30) within the top 30m of the soil. 2.Near-field pulse abbreviated as NF; Near-field non pulse abbreviated as NFN; Far-field abbreviated as FF. The fault distance of near-field earthquakes is less than 20 km, while that of far-field earthquakes is more than 20 km, the division of fault distance is based on Reference [1].**

**Table 2:** The CV of PGA for strong motion records.

Site Classes	Fault Distance	CV
Class I (Vs30 > 510m/s)	NF	0.74
	NFN	0.95
	FF	0.89
Class Ⅱ (260m/s < Vs30 ≤ 510m/s)	NF	0.85
	NFN	0.83
	FF	0.87
Class Ⅲ (150m/s < Vs30 ≤ 260m/s)	NF	0.88
	NFN	0.83
	FF	0.86

## 3. The elastic-plastic displacement spectra

**Fig 2:**
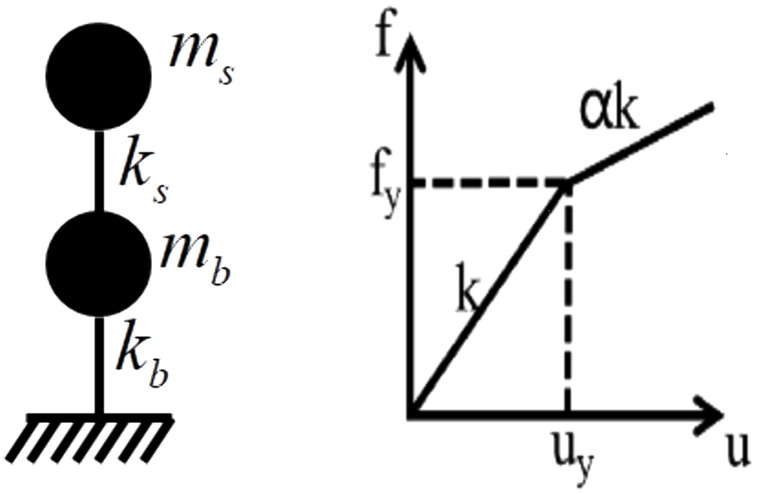
The analytical model for the elastic-plastic response spectra. **(a)** The analytical model, **(b)** The bilinear force-deformation relationship.

The horizontal stiffness of the isolation bearing is significantly lower compared to the superstructure, resulting in the upper structure behaving as a rigid body under seismic action, with deformation primarily localized in the isolation layer. Hence, a base-isolated structure can be approximated as a two-degree-of-freedom system. The analytical model and the bilinear force-deformation relationship of the isolation bearing are illustrated in Fig 2.

The motion equation of a double-degree freedom system under seismic action:


$$M\ddot{X}+C\dot{X}+F=-MI{\ddot{x}}_{g}$$ (1)

where, M, C represent the mass matrix and damping matrix of the system, respectively. Herein, the non-proportional damping is employed, the damping ratio of the upper structure is 0.05, the equivalent damping ratios of the isolation layer are 0.10, 0.20, 0.30, 0.40, because the damping ratio between 0.05 and 0.40 is comforted the actual requirements of the base-isolated structures [36]; 𝐈 is the column vector of earthquake location, each element is 1; $\dot{X}$, $\ddot{X}$ represent the relative velocity and relative acceleration vectors of the system, respectively; ${\ddot{x}}_{g}$ is the seismic acceleration; F is the resilience of the system, the Bouc-wen model is used to describe the bilinear force-deformation behavior of isolation bearings, which is described in Equation (2).


$$F=KX+H(1-\alpha )F_{y}Z$$ (2)

where, α is the stiffness ratio of the isolation bearings after yielding to before yielding, which is taken as 0.07 [20–21]; K is the stiffness matrix of the system; X is the relative displacement vector of the system; H is a column vector representing the position of dimensionless components of the isolation layer. Note that $H={\left[1,0\right]}^{T}$. $F_{y}=k_{b}x_{y}$ is the yield force of the isolation bearings; Z is the dimensionless component of the isolation layer considering the material hysteresis characteristics, which is shown as:


$$x_{y}\dot{Z}=A{\dot{x}}_{b}-\gamma \left|{\dot{x}}_{b}\right|Z{\left|Z\right|}^{\eta -1}-\beta {\dot{x}}_{b}{\left|Z\right|}^{\eta }$$ (3)

where $x_{y}$ is the yield displacement of the isolation bearing, ${\dot{x}}_{b}$ is the relative velocity of the isolation bearing, A, γ, β, and η are the dimensionless parameters of the hysteresis loop, A, γ, β, η, and α control the shape of the hysteresis loop, these parameters are usually determined by experiments, note that β+γ=1. Herein, both A and η are 1, both γ and β are 0.5 [37–38].

The displacement spectra is the peak displacement of the isolation layer under a given seismic action with respect to the natural vibration period of the system:


$$S_{d}=\max\left|x(t)\right|$$ (4)

**Fig 3:**
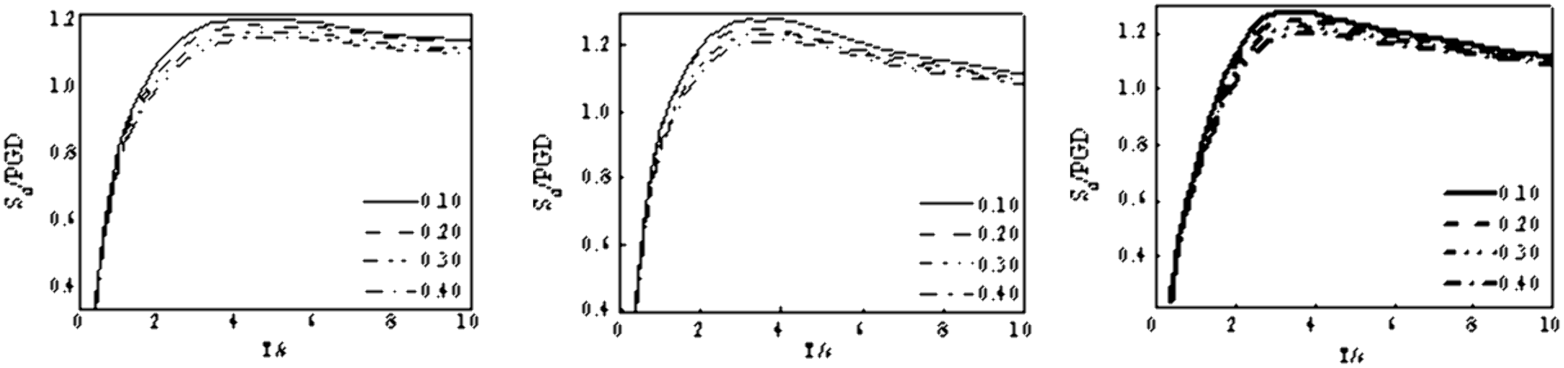
Displacement response spectra. (a) Class Ⅰ, (b) Class Ⅱ, (c) Class Ⅲ.

Two methods are typically employed to establish the displacement spectra. The first determine the seismic ground motion of the building site through seismic hazard analysis. Then, a large number of nonlinear dynamic response analysis results is collected to produce the displacement spectra. The other method converts the acceleration spectra from existing codes into displacement spectra. This paper adopts the first method, and the elastic-plastic response of a double degree of freedom system is calculated using the MATLAB. The analysis period range is chosen from 0.4 s to 10 s because the natural period of base-isolated structures is longer than traditional structures. Fig 3 shows the displacement spectra of the isolation layer.

## 4. Sensitivity of displacement spectra

The displacement spectra are essential for the design of base-isolated structures. To map the displacement spectra, the influences of site classification, earthquake type, and parameter in Bouc-wen model on the displacement spectra are investigated in this section.

### 4.1. Site condition

**Fig 4:**
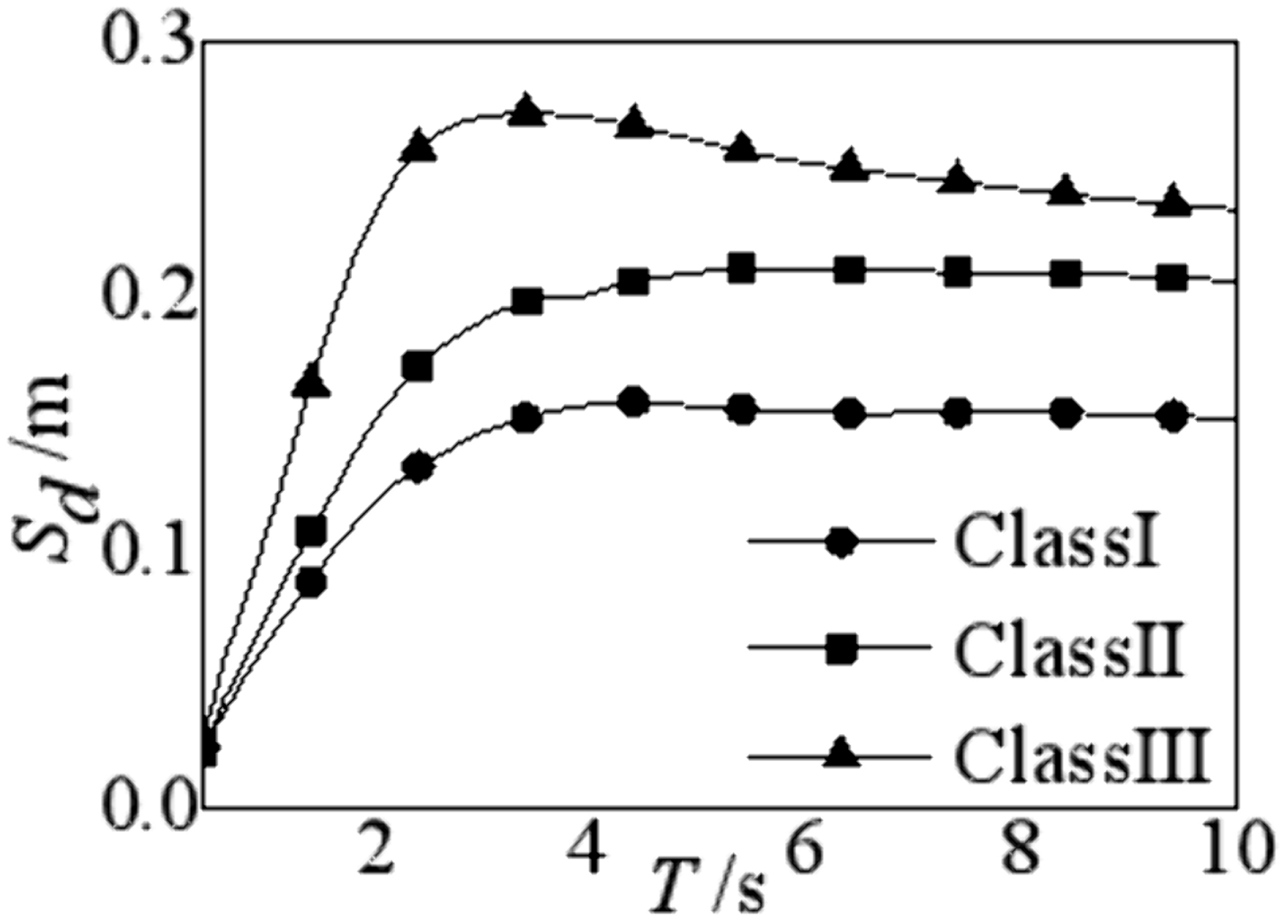
Displacement response spectra in different site conditions.

Site conditions are closely related to the elastic-plastic response of structures [39–43]. To study the influence of site classification on the displacement spectra, 1217 strong motion records are employed to perform the elastic-plastic analysis on the base-isolated structures. Accordingly, the average displacement spectra for the three site conditions are shown in Fig 4.

Fig 4 demonstrates the displacement spectra for base-isolated structures show similar trends with varying periods. The displacement response increases rapidly with the period increasing. However, once a specific threshold is reached, the curve of displacement spectra commences to decline and subsequently plateaus. The transition from site condition I to III results in an augmented displacement within the isolation layer for base-isolated structures with identical periods. Thus, the softer soil produces the greater spectral value of the displacement response. Within the range of natural periods of base isolated structures (approximately 1.5s to 4.0s), the ratio of the maximum displacement response at Site III to Site I is 1.56, while the ratio of Site III to Site II is 1.46. Hence, the influence of site conditions must be considered in the seismic design of base isolated structures.

### 4.2. Earthquake type

**Fig 5:**
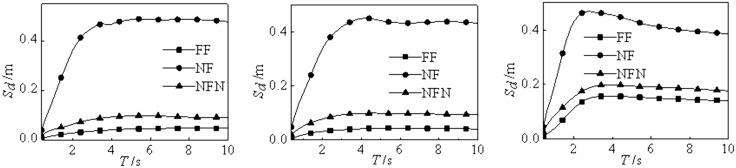
Displacement response spectra in different earthquake types. (a) ClassⅠ, (b) Class Ⅱ, (c) Class Ⅲ.

The near-field ground motion is marked by significant long-period velocity and displacement pulses. These pulses can directly impart high-energy impacts on structures, resulting in large displacements and deformations of buildings [44–48]. Table 1 categorizes strong motion records based on earthquake types,the elastic-plastic response of the base-isolated structure under various site conditions and earthquake types is calculated to obtain the displacement spectra, as shown in Fig 5. Fig 5 illustrates the displacement response spectra for the three types of strong motion records are similar under the same site conditions. Note that the displacement response of the isolation layer in base-isolated structures is more significant than that under near-field non-pulse-type and far-field earthquakes. In addition, the displacement under the near-field pulse-type earthquake action is approximately 11.38 times than the far-field earthquake action. Therefore, this crucial factor must be taken into utmost consideration by designers during the design process.

### 4.3. Parameters in Bouc-wen model

**Fig 6:**
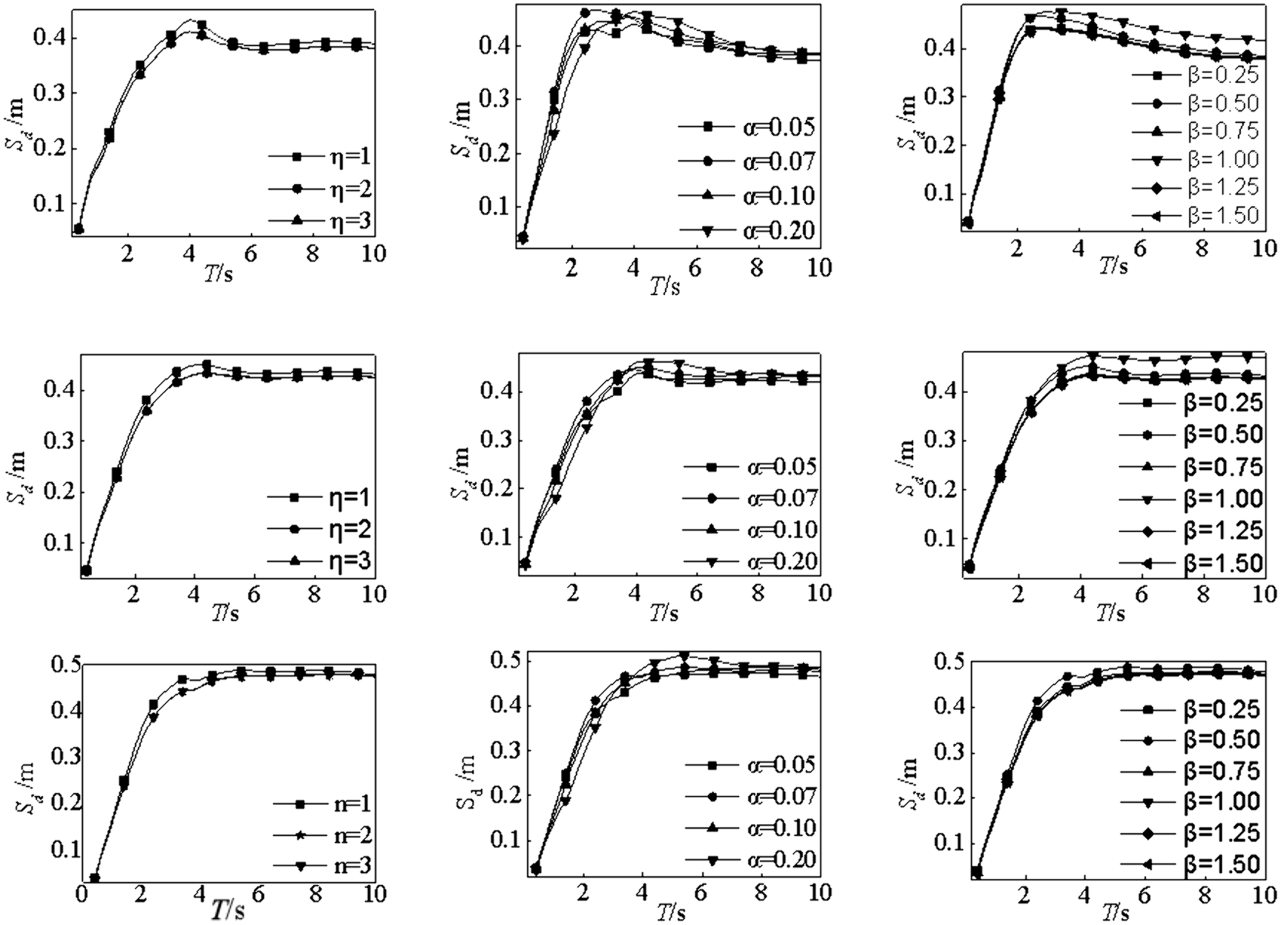
Displacement response spectra in different parameters of Bouc-wen model. (a) ClassⅠ, (b) Class Ⅱ, (c) Class Ⅲ.

According to Equations (1)-(3), the parameters of Bouc-wen model, may have effects on the base-isolated layer response of base-isolated structures. The parameters of Bouc-wen model varies in a certain range. To study the effect of parameters of Bouc-wen model on base-isolated structure [38,49], the range of η is 1 ~ 3; the range of α is 0.05 ~ 0.20; and the range of β is 0.25 ~ 1.5. According to Section 2.2, the response of base-isolated layer is the largest under the action of near-field pulse ground motion for all site conditions, Thus, 118 near-field pulse ground motion strong motion records are employed to perform elastic-plastic analysis on base-isolated structures. The average displacement spectra for the three site conditions are shown in Fig 6.

The displacement spectra under different parameters of Bouc-wen model are similar. Thus, the displacement spectra is less influenced by the parameters of Bouc-wen model. Note that the softer soil on the site would induce larger spectral value of the displacement response. In addition, the spectral value of the displacement spectra will decrease with the increase of the parameters of the Bouc-wen model. The maximum influence of η on the displacement spectrum is as follows: when η = 3, the displacement spectra of site Ⅲ is 1.13 times that of the site I; The maximum influence of α on the displacement spectrum is as follows: when α = 0.20, the displacement spectra of site Ⅲ is 1.31 times that of the site I; The maximum influence of β on the displacement spectrum is as follows: when β = 1.50, the displacement spectra of site Ⅲ is 1.32 times that of the site I. Accordingly, the effect of Bouc-wen model parameters on the displacement response spectra is negligible compared with that of site conditions and earthquake types.

## 5. Displacement response spectrum modeling and its application

### 5.1. Mapping of the displacement response spectrum

The displacement response spectrum is mainly affected by site category and earthquake type. Thus, this study maps the relationship between the displacement spectra and site category and earthquake type. Note that the actual seismic design requires representative displacement spectra, herein, the displacement spectra is standardized and averaged. The displacement spectrum is first normalized according to the peak ground motion. Then, the mean within the group is calculated. After considering the effects of site category and earthquake type, the displacement response spectrum can be mapped as


$$\begin{array}{c} \frac{S_{d}}{PGD}=\left\{ \begin{array}{cc} 0.472\ln T+0.812 & 0.4\leq T<2.0\\ -0.038(T-2{)}^{2}+0.122(T-2)+1.138 & 2.0\leq T<4.0\\ -0.021(T-4{)}^{2}+1.23 & 4.0\leq T<10.0 \end{array}\right. \end{array}$$ (5)

**Fig 7:**
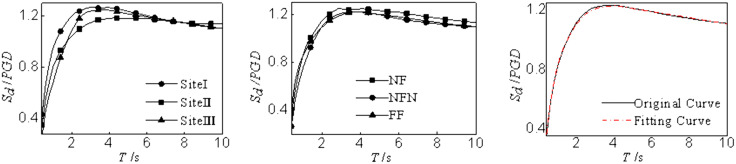
Modeling of displacement spectra. (a) Averaging earthquake types, (b) Averaging site classes, (c) Averaging earthquake types and site classes.

The relationships between the displacement spectra and site category and earthquake type are shown in Fig 7. According to the average response spectrum, the displacement response of the structure changes rapidly with the natural period when the structural natural period is short; conversely, the displacement response changes slowly and reaches a peak with the change of the natural period when the structural natural period is long. In addition, when the structural natural period is exceptionally long, the displacement response becomes invariant with further increases in the natural period, ultimately converging to a constant value. As illustrated in Fig 7(c), the fitted expression accurately captures and represents the modeled displacement spectrum.

### 5.2. Application of the displacement spectrum

The horizontal stiffness of the isolation layer is small, excessive deformation generated during a strong motion record cause the decrease in the vertical bearing capacity of the isolation bearings or even collapse of the isolated structures. Therefore, the displacement of the isolation layer must be controlled within an allowable range in the seismic design.

Equation (5) represents the modeled displacement spectrum, however, the effect of site conditions is necessary when using this Equation to calculate the displacement response of the structure. Therefore, the average of PGD/PGA for the three site categories are calculated using the selected 1217 strong motion records, as listed in Table 3. The average values in the table represent the influence of site category on the structural displacement. Accordingly, the maximum earthquake acceleration for each design intensity level (As shown in Table 4) provided by the code is used. The peak ground displacement (PGD) corresponding to each category of site conditions can be obtained, as shown in Table 5.

**Table 3:** Mean ratios of PGD and PGA.

	Earthquake Type	Site Ⅰ	Site Ⅱ	Site Ⅲ
*PGD/PGA*(s^2^)	NF	0.106	0.115	0.122
	NFN	0.040	0.049	0.058
	FF	0.032	0.033	0.036

**Table 4:** The maximum acceleration for the earthquake in time history analysis.

Seismic precautionary intensity	6	7	8	9
Frequent earthquake	18	35 (55)	70 (110)	140
Precautionary eaarthquake	50	100 (150)	200 (300)	400
Rare earthquake	125	220 (310)	400 (510)	620

**Table 5:** Design peak ground displacement at different sites (Unit: cm).

Condition			Seismic precautionary intensity			
			6	7	8	9
Ⅰ	Frequent earthquake	NF	1.903	3.701 (5.816)	7.402 (11.631)	14.804
		NFN	0.716	1.392 (2.187)	2.784 (4.375)	5.568
		FF	0.591	1.149 (1.805)	2.297 (3.610)	4.595
	Precautionary eaarthquake	NF	5.287	10.574 (15.861)	21.148 (31.722)	42.296
		NFN	1.989	3.978 (5.966)	7.954 (11.931)	15.908
		FF	1.641	3.282 (4.923)	6.564 (9.846)	13.128
	Rare earthquake	NF	13.218	23.268 (32.779)	42.296 (53.927)	65.559
		NFN	4.971	8.749 (12.329)	15.908 (20.283)	24.657
		FF	4.102	7.220 (10.174)	13.128 (16.738)	20.348
Ⅱ	Frequent earthquake	NF	2.063	4.011 (6.304)	8.023 (12.607)	16.045
		NFN	0.884	1.719 (2.701)	3.438 (5.402)	6.875
		FF	0.598	1.162 (1.826)	2.324 (3.652)	4.648
	Precautionary eaarthquake	NF	5.731	11.461 (17.192)	22.922 (34.383)	45.844
		NFN	2.456	4.911 (7.367)	9.822 (14.733)	19.644
		FF	1.660	3.320 (4.980)	6.640 (9.960)	13.280
	Rare earthquake	NF	14.326	25.214 (35.529)	45.844 (58.451)	71.058
		NFN	6.139	10.804 (15.224)	19.644 (25.046)	30.448
		FF	4.150	7.304 (10.292)	13.280 (16.930)	20.584
Ⅲ	Frequent earthquake	NF	2.191	4.261 (6.696)	8.522 (13.391)	17.044
		NFN	1.049	2.040 (3.206)	4.080 (6.412)	80161
		FF	0.653	1.269 (1.995)	2.539 (3.990)	5.078
	Precautionary eaarthquake	NF	6.087	12.174 (18.261)	24.348 (36.522)	48.696
		NFN	2.914	5.829 (8.744)	11.658 (17.487)	23.316
		FF	1.814	3.627 (5.441)	7.254 (10.881)	14.508
	Rare earthquake	NF	15.218	26.783 (37.739)	48.696 (62.087)	75.479
		NFN	7.286	12.824 (18.070)	23.316 (29.728)	36.140
		FF	4.534	7.979 (11.244)	14.508 (19.498)	22.487

During the design of base-isolated structures, the natural period *T* for a given base-isolated structure is determined and can be calculated using Equation (5). In addition, the ratio of the structural displacement to PGD (referred to as displacement amplification factor) under seismic action can be obtained from Table 5. Accordingly, the maximum displacement response of the base-isolated structure under a specific seismic intensity for a certain category of site conditions is determined. Herein, the maximum displacement obtained is considered as the allowable displacement for the isolation layer of the base-isolated structure.

## 6. Case study

### 6.1. Numerical model

The influence factors of displacement response spectra of base-isolated structure are introduced. In addition, and the mapping of displacement response spectra is summarized. Taking two reinforced concrete frame structures with an 8-story and a 7-story superstructure as examples, the rationality of the formula is verified.

The parameters of the 8-story structure are as follows [37]: The mass of each floor of the superstructure is 5 × 10^5 kg^, and the stiffness of each floor is 2.3 × 10^8^N/m; the mass of the isolation layer is 3.3 × 10^5 kg^, the total stiffness of the isolation layer is 1.92 × 10^5^N/m., and the natural vibration period is 2.24 s.

The parameters of the 7-story structure are as follows [50]: The mass of each floor of the superstructure is 54.99 × 10^5 kg^, 52.66 × 10^5 kg^, 53.13 × 10^5 kg^, 50.52 × 10^5 kg^, 31.11 × 10^5 kg^, 7.08 × 10^5 kg^, respectively. The stiffness of each flooris 97.96 × 10^8^N/m, 51.41 × 10^8^N/m, 43.25 × 10^8^N/m, 33.87 × 10^8^N/m, 28.58 × 10^8^N/m, 21.75 × 10^8^N/m, and 17.98 × 10^8^N/m, respectively. The mass of the isolation layer is 75.48 × 10^5 kg^, the horizontal stiffness of the isolation layer is 4.19 × 10^8^N/m, and the natural vibration period is 1.98s.

**Fig 8:**
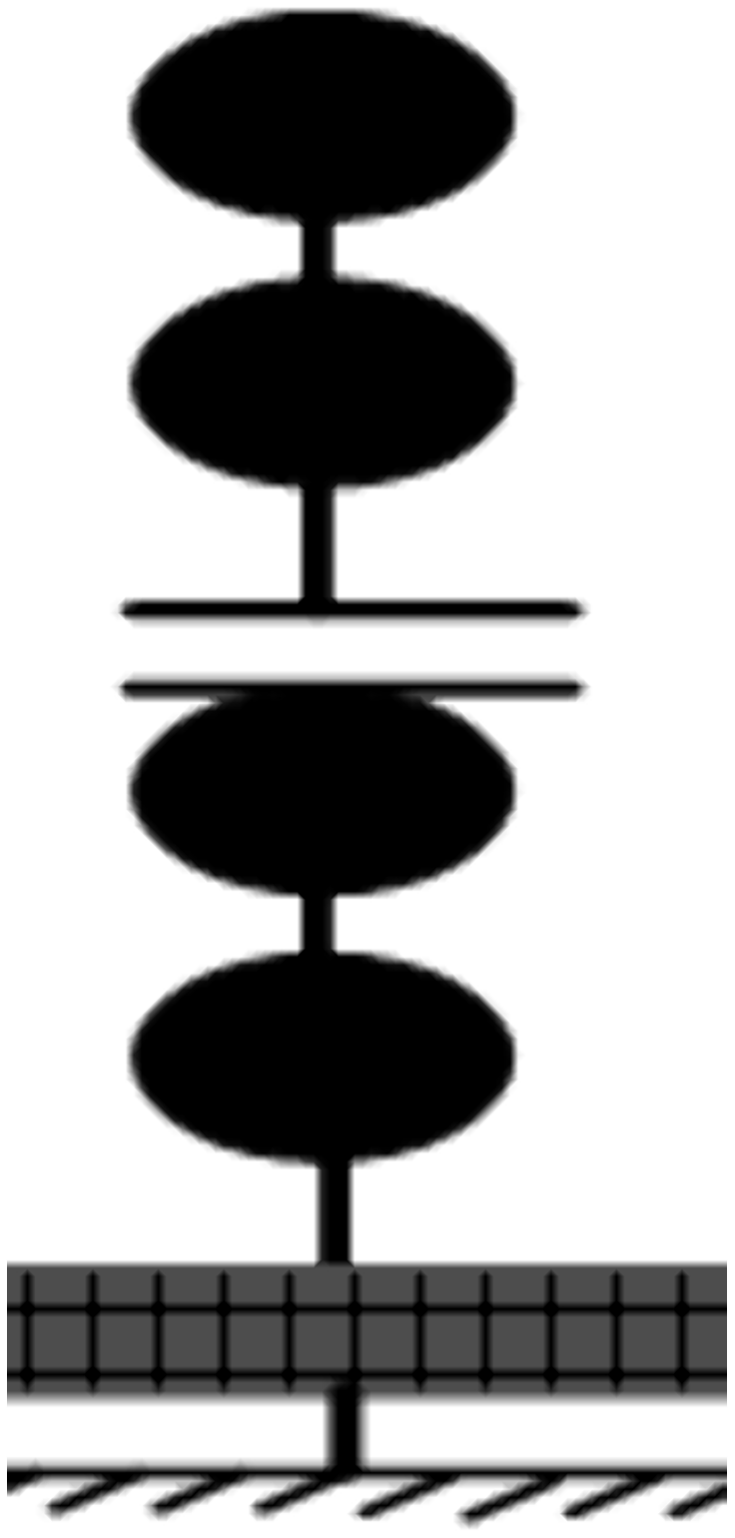
The calculation model.

Fig 8 shows the calculation model of the base-isolated structures.

### 6.2. Verification of the molded displacement spectra

The displacement of base-isolated layer is obtained using the time-history analysis method by MATLAB. The calculations of class II are taken as an example. To verify the applicability of the response spectrum proposed in this paper, when selecting ground motion records, the spectrum matching method specified in Reference [1] was adopted. Specifically, at the period points corresponding to the main vibration modes of the structure, the error between the average seismic influence coefficient curves of multiple groups of ground motion records and the target spectrum is less than 20%. Herein, the target spectrum refers to the elastoplastic displacement spectrum of the isolation layer proposed in this study. Herein, 21 strong motion records of different earthquake types are selected, the detailed information is shown in Table 6. Note that the selected RSN numbers are listed since all the strong motion records in PEER have RSN numbers.

**Table 6:** Strong motion records.

RSN number	Earthquake type	Site Classes
147、766、767、1004、1086、1119、3475	NF	Ⅱ
139、154、230、585、1083、1194、4121	NFN	
17、65、83、94、121、166、937	FF	

**Fig 9:**
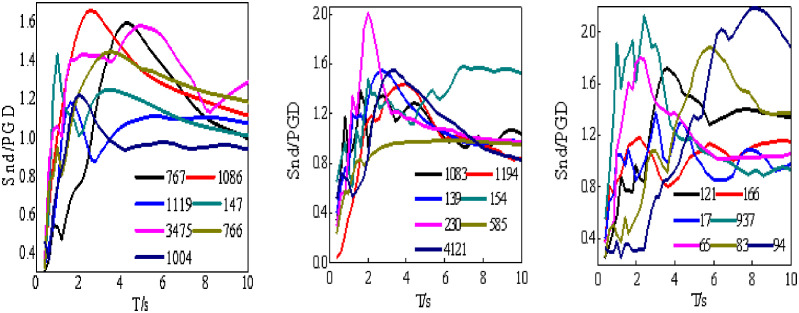
The nonlinear displacement spectra of strong motion records. (a) NF, (b) NFN, (c) FF.

**Fig 10:**
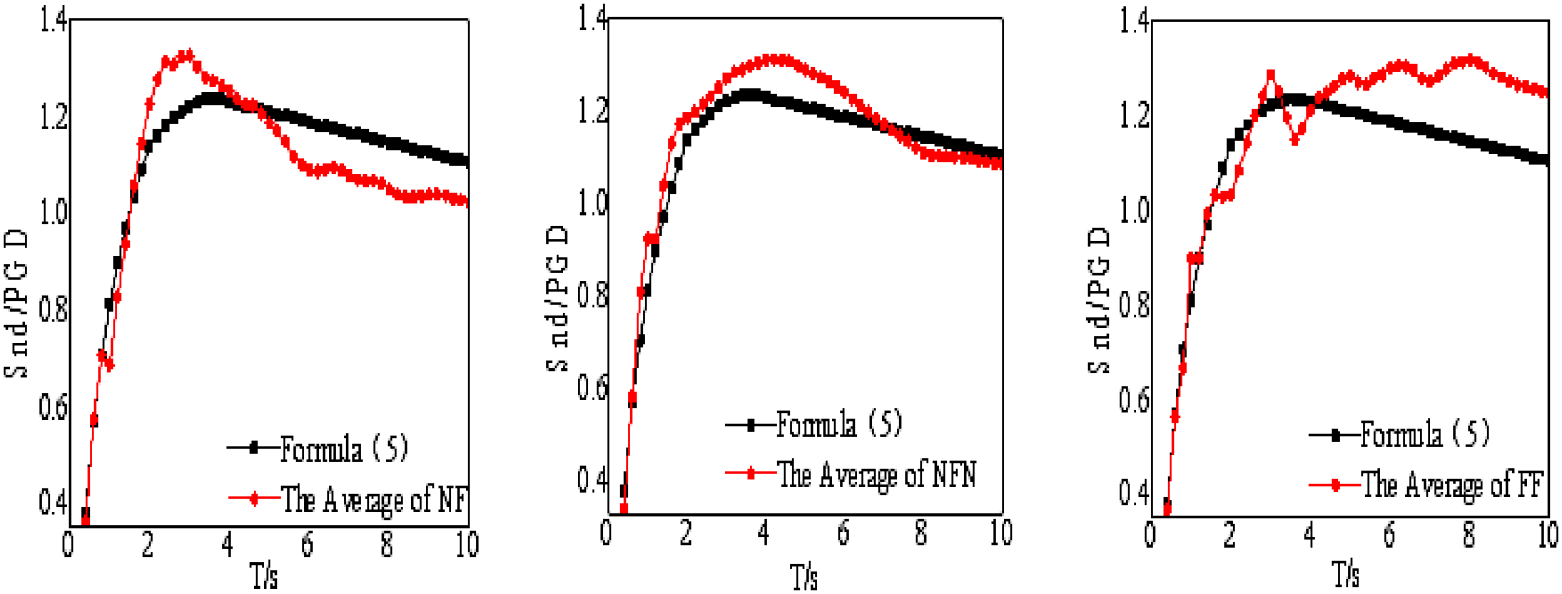
The average spectrum and the nonlinear displacement spectrum of the modeled isolation layer. (a) NF, (b) NFN, (c) FF.

The comparison between the spectrum of strong motion records for time-history analysis, the average spectrum, and the nonlinear displacement spectrum of the modeled isolation layer, as well as the errors between the average spectrum and the displacement spectrum of the modeled isolation layer, are shown in Figs 9 and 10, respectively.

**Fig 11:**
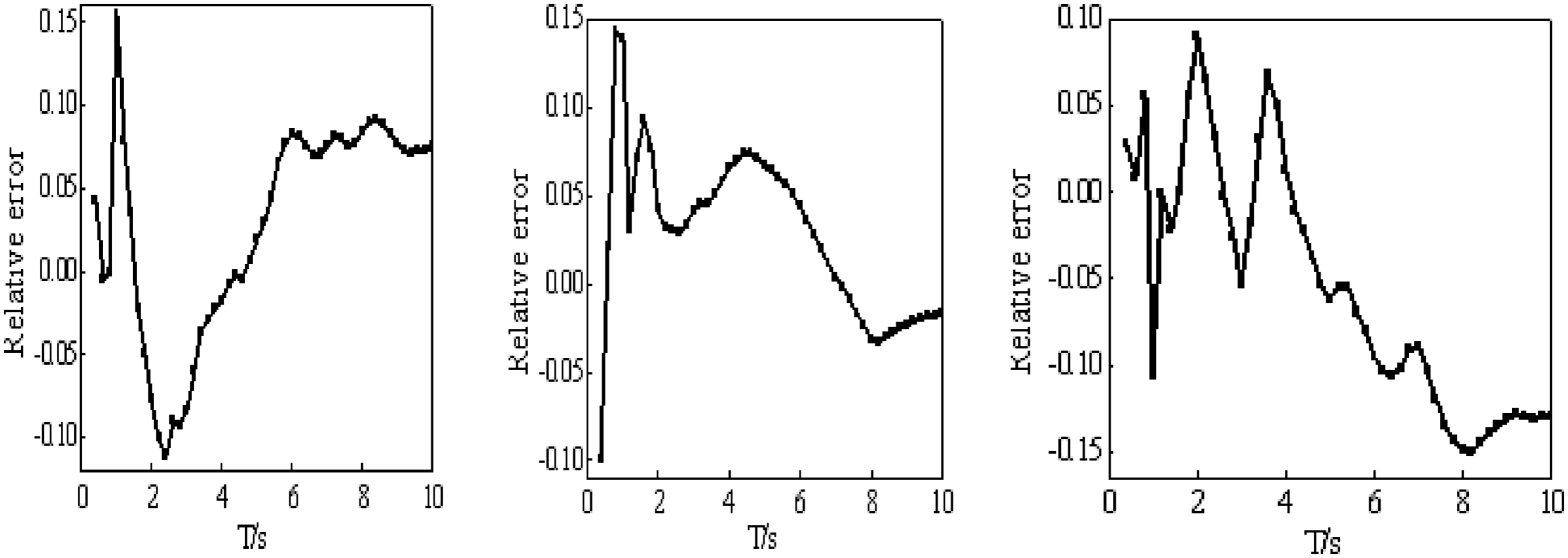
The relative error of average spectrum and modeled spectrum. (a) NF, (b) NFN, (c) FF.

As shown from Fig 11, the relative error between the average spectrum of near-field pulse earthquakes and the modeled displacement spectrum ranges from −9.9% to 14.4%, that of near-field non-pulse earthquakes ranges from −11.1% to 15.5%, and that of far-field earthquakes ranges from −14.9% to 9.0%. Therefore, the absolute values of the relative errors between the average spectra and the nonlinear displacement spectra of the modeled isolation layer are all less than 20% across the entire period range, strictly meeting the requirements of the code.

The 8 degree (0.2g) is utilized as an example to verify the accuracy of the modeled displacement spectra, the selected 21 strong motion records are modulated according to frequent (0.07g), precautionary (0.20g) and rare (0.40g), The displacement response of isolation layer is calculated and compared with the displacement response of the isolation layer obtained according to Equation (5), as demonstrated in Table 7.

**Table 7:** Displacement of the isolated layer (Unit: m).

Condition			T = 2.24s			T = 1.98s		
			Formula calculation	Time history analysis	Error/%	Formula calculation	Time history analysis	Error/%
Ⅱ	Frequent earthquake	NF	0.093	0.092	0.83	0.084	0.099	17.79
		NFN	0.040	0.040	0.09	0.032	0.036	11.82
		FF	0.027	0.024	11.81	0.026	0.023	−15.57
	Precautionary eaarthquake	NF	0.267	0.265	0.66	0.240	0.233	−2.99
		NFN	0.114	0.113	1.52	0.090	0.096	6.19
		FF	0.077	0.074	4.16	0.074	0.076	1.77
	Rare earthquake	NF	0.534	0.561	−4.83	0.480	0.471	−1.94
		NFN	0.229	0.245	−6.72	0.180	0.164	−10.39
		FF	0.155	0.172	−9.81	0.149	0.144	−3.45

Table 7 shows the maximum displacements of the isolation layer, which are produced by the response spectra and the time history analysis method. Herein, these maximum displacements are determined by the structural period, the average ratio of the peak ground motion, and the peak displacement of the ground motion. Since the response spectra consolidate the characteristics of numerous seismic waves, while the time history analysis method relies on several strong earthquake records, the results from both methodologies are inherently subject to certain deviations. Nonetheless, as evident from Table 6, all errors are contained within 20%, indicating that the values obtained through the fitting expression adequately capture the displacement response of the isolated structure. Thus, the response spectra provide a basis for estimating the response of base-isolated structures.

## 7. Conclusion

This study examined the impact of site conditions, earthquake types, and Bouc-Wen model parameters on the displacement response spectrum of bilinear two-degree-of-freedom systems with base-isolated structures. Herein, 1217 strong motion records are employed. By averaging the two influencing factors, the modeled displacement spectrum was derived and subsequently validated using two base-isolated structures with different natural periods. The following conclusions are drawn:

1) Three factors affect the displacement of base-isolated structure, among them, the effect degree of site is: Site III > Site II > Site I; the effect degree of earthquake type is: NF > NFN > FF; the effect degree of the Bouc-wen model parameters is β > α > η. Because the influence of Bouc-wen model parameters is smaller than the two others, the site type and earthquake type should be considered in the seismic design of base-isolated structures.
2) The displacement spectrum was standardized and averaged, accordingly, a three-segment modeled displacement spectrum is mapped. The resulting fitting expression effectively encapsulates and represents the characteristics of the modeled displacement spectrum.
3) The proposed modeled displacement spectrum was verified using the time history analysis, which can reliably predict the displacement of the isolation layer in base-isolated structures across diverse site conditions and earthquake impacts. Moreover, during the process of intelligent construction, it enables more accurate seismic performance evaluation of the structures.
4) Only a limited range of Bouc-Wen model parameters were considered, not covering all nonlinear hysteretic behaviors, temperature effects and material aging on bearing performance were not considered, and the influence of vertical ground motions on the isolation layer was not analyzed. Next, research will be carried out on expanding parameter ranges, considering three-dimensional ground motion input, and optimizing isolation layer design methods.

## Supporting information

S1 File Computational Program and Classified Earthquake Records. (RAR)
